# Pathophysiological effects of SARS-CoV-2 infection on the cardiovascular system and its clinical manifestations—a mini review

**DOI:** 10.3389/fcvm.2023.1162837

**Published:** 2023-05-16

**Authors:** Juan Carlos Yugar-Toledo, Louise Buonalumi Tacito Yugar, Luis Gustavo Sedenho-Prado, Roberto Schreiber, Heitor Moreno

**Affiliations:** ^1^ Medicine Department, Faculty of Medicine of São José do Rio Preto (FAMERP), São Paulo, Brazil; ^2^ School of Medical Sciences, State University of Campinas (UNICAMP), São Paulo, Brazil; ^3^ Department of Internal Medicine, School of Medical Sciences, State University of Campinas (UNICAMP), São Paulo, Brazil

**Keywords:** SARS-CoV-2, cardiovascular disease, myocarditis, heart failure, arrhythmia, cardiogenic shock

## Abstract

Coronavirus disease 2019 (COVID-19) is a viral infection caused by severe acute respiratory syndrome coronavirus 2 (SARS-CoV-2). COVID-19 may have a mild presentation, with few symptoms, or progress to a severe condition, characterized by generalized inflammation, systemic microvascular involvement, coagulopathy, and pulmonary and cardiovascular complications. Men present with more severe symptoms than women, especially men who are older and who present with comorbidities such as hypertension, diabetes mellitus, and a history of atherosclerotic diseases. Owing to its association with endothelial dysfunction, inflammation, thrombosis, and microvascular obstruction, SARS-CoV-2 infection can cause lesions in several organs, including the myocardium and the coronary arterial bed, which can result in clinical manifestations involving the cardiovascular system. In this mini review, we summarize the effects of SARS-CoV-2 infection on the cardiovascular system in both children and adults and characterize the various clinical manifestations associated with this disease.

## 1. Introduction

The coronavirus disease 2019 (COVID-19) pandemic, caused by severe acute respiratory syndrome coronavirus 2 (SARS-CoV-2), resulted in extensive extrapulmonary manifestations of the disease. These manifestations are the result of inflammatory processes involving multiple organs, resulting in the release of immune signaling mediators such as cytokines, tumor necrosis factor-alpha (TNF-α), and interleukin (IL)-1 and -6. These immune response mediators affect the cardiovascular system as a whole and can lead to abnormal coagulation and thromboembolic events (1, 2).

Acute myocardial infarction, acute coronary syndrome, cerebral vascular accidents, peripheral obstructive arterial disease with a risk of limb amputation, venous system involvement with deep vein thrombosis, and pulmonary thromboembolism are some of the cardiovascular events resulting from SARS-CoV-2 infection. Other complications include ventricular cardiac arrhythmias, supraventricular tachycardia [including atrial fibrillation (AF)], atrioventricular blocks, and direct myocardial injury, which can lead to myocarditis, heart failure, and cardiogenic shock (3–5).

## 2. Pathophysiology of the cardiovascular manifestations of SARS-CoV-2

Several viral infections cause heart failure because of direct viral invasion and a storm of proinflammatory cytokines, leading to the activation of the sympathetic system and to myocardial failure. Such inflammatory overload, particularly the elevation of IL-1β, IL-6, and monocyte chemoattractant protein-1, leads to fulminant myocarditis. Moreover, endothelial dysfunction associated with multisystem inflammation and decreased nitric oxide bioavailability contribute to heart failure. These symptoms can also result from a combination of preexisting heart disease and virus-related acute hemodynamic and hypoxemic stress.

Viruses enter the cytoplasm of host cells, like myocardial cells, after the host and viral membranes fuse, following cleavage of the viral S protein by transmembrane protease, serine 2. SARS-CoV-2 uses the spike protein to bind to angiotensin-converting enzyme 2 (ACE2) receptors on the myocardial cell membrane (6), which triggers the negative regulation of these receptors, angiotensin II accumulation, and subsequent adverse myocardial remodeling through the activity of angiotensin II type 1 receptors (7). SARS-CoV-2 can also cause myocardial damage via cell-mediated cytotoxicity. This uses a positive-feedback loop mechanism, in which activated CD8+ T lymphocytes migrate to cardiomyocytes and cause myocardial inflammation. Proinflammatory cytokines released into the bloodstream promote T-cell activation, which results in increased cytokine release (8).

SARS-CoV-2 can affect the myocardium through three different mechanisms: (1) direct myocardial injury caused by the entry of the virus coupled to ACE2 receptors, which induces inflammation and cardiomyocyte death; (2) indirect secondary damage caused by a downregulation of ACE2 expression during postviral replication, which results in a hyperactivation of the renin–angiotensin system and stimulation of angiotensin 1 receptors, thereby promoting inflammatory and oxidant activities and arterial vasoconstriction; (3) indirect action mediated by immune B- and T-cell activation, resulting in a systemic inflammatory response with increased oxidative stress and an imbalance between oxygen supply and consumption (9, 10).

Inflammation is a major characteristic of these different damaging mechanisms induced by SARS-CoV-2 infection in the myocardium. Elevated ILs (including IL-2, -7, and -10), TNF-α, and the above-mentioned cytokine storm cause a negative inotropic effect and contribute to myocardial injury, apoptosis, and fibrosis. Furthermore, macrophage activation results in the release of IL-1 and IL-6, which promote inflammatory cell infiltration, vascular injury, microvascular involvement, endothelial dysfunction, and expression of cell adhesion molecules, including intercellular adhesion molecule 1 and vascular cell adhesion molecule 1 (11–15).

Therefore, SARS-CoV-2 infection is associated with an increased risk of infarction and acute coronary syndrome (16, 17). T-cell hyperactivation has been shown to induce the production of large amounts of interferon, TNF-α, and IL-6, which promotes immune system dysregulation and vascular diseases such as atherosclerosis (18). Increases in D-dimer levels and changes in fibrinolytic mechanisms, such as the inhibition of antithrombin of protein C and tissue factor, can lead to coronary thrombosis at the sites of plaque rupture and prothrombotic conditions in SARS-CoV-2-related inflammation (19).

Supplementary Material Figure S1 shows the pathophysiology of cardiovascular manifestations resulting from SARS-CoV-2 infection and effects on the cardiovascular system.

Some children with SARS-CoV-2 infections experience inflammatory shock, similar to that in Kawasaki disease, whose symptoms include heart failure and coronary artery disease (20–23). An interaction has been suggested between the hyperinflammatory state [caused by the cytokine storm (24) in monocytes] and the activated macrophages, characteristic of both Kawasaki disease and SARS-CoV-2 (25).

In Kawasaki disease, cell infiltration is initiated by lymphocytes and macrophages in the tunica intima and adventitia (6–8 days after the onset of clinical symptoms). This cell infiltration process then progresses to the rest of the arterial wall (at approximately 10 days after onset) and leads to coronary artery involvement (arteritis). The extracellular matrix, which contains elastin, is required for maintaining the structural integrity of the arterial wall; its degradation by matrix metalloproteinases contributes to vascular inflammation (26). The inflammatory process in Kawasaki disease progression involves leukocytes, macrophages, lymphocytes (27), and high levels of TNF-α, a primary factor in this disease.

Conversely, SARS-CoV-2 causes endothelial cell infection and inflammation (endotheliitis) (28–30), which promotes microcirculatory dysfunction and endothelial cell apoptosis, and can be a contributing factor to the development of arteritis in Kawasaki disease. The inflammatory response to severe SARS-CoV-2 infection is potentiated by interferon-1 (31, 32). An elucidation of the molecular mechanisms associated with the inflammatory response activation could shed light on the pathogenesis of both Kawasaki disease and COVID-19.

## 3. Cardiovascular manifestations of SARS-CoV-2 in adults

### 3.1. Acute coronary artery disease and SARS-CoV-2

SARS-CoV-2 infection causes extrapulmonary symptoms, such as acute myocardial infarction or acute coronary syndrome, which affect 1.7% (confidence interval, 0%–3.6%) of patients hospitalized for the virus. Despite the majority of these patients having no history of coronary heart disease, many present with ST-segment elevation myocardial infarction, which is caused by the rupture of vulnerable coronary atherosclerotic plaques, coronary spasm, endothelial dysfunction, and thrombosis. Nevertheless, the number of hospitalizations for acute coronary events and percutaneous interventions registered in the United States, Italy, and Spain during the SARS-CoV-2 pandemic was significantly lower than the preceding period (33–35), possibly because fewer people sought medical care for heart attacks (36).

SARS-CoV-2 infection can contribute to an increase in plasma troponin levels above the 99th percentile, which is indicative of myocardial injury. The major type of injury associated with SARS-CoV-2 is acute myocardial infarction, caused by acute myocardial ischemia, which can be of two types. Type 1 infarction typically results from plaque rupture, ulceration, erosion, dissection, and thrombosis; conversely, type 2 infarction is caused by an imbalance between oxygen supply and demand in heart muscles. To treat this condition, it is crucial to determine the type of infarction (37), both of which can occur in patients infected with SARS-CoV-2 (38). The presence of comorbidities, such as diabetes mellitus, arterial hypertension, and obesity, partially explains the high prevalence of coronary events in patients with COVID-19, as well as the higher incidence in severe cases.

### 3.2. Takotsubo syndrome and SARS-CoV-2

Similar to myocardial injury, Takotsubo syndrome is associated with increased plasma troponin levels. In this syndrome, the segmental contractility of the left ventricular wall is altered, resulting in hypokinesia and akinesia, which lead to sudden heart failure and, in rare cases, can mimic acute myocardial infarction. Although its etiology is unclear, sympathetic hyperstimulation with microvascular involvement caused by stress-induced catecholamines appears to be an underlying cause (39). Takotsubo syndrome is also associated with high-grade inflammation (40–42).

### 3.3. Acute myocardial involvement (myocarditis) and SARS-CoV-2

Acute myocardial involvement is the most common cardiac complication associated with SARS-CoV-2 infection. Typical symptoms include acute heart failure (3%–33%), left ventricular dysfunction (10%–41%), right ventricular dysfunction (33%–47%), and biventricular dysfunction (3%–15%) (43, 44); presence of electrocardiogram (ECG) abnormalities and increased cardiac enzymes, such as high-sensitivity cardiac troponin T (hs-cTn) and N-terminal B-type natriuretic peptide (NT-proBNP) are indicators of these clinical complications (45).

Ventricular wall stress caused by pressure or volume overload is the main stimulus for natriuretic peptide synthesis and release, which act in the kidney, inducing natriuresis and diuresis. Other physiological effects include peripheral vasodilation and inhibition of the renin–angiotensin and sympathetic nervous systems. NT-proBNP has a half-life of 120 min and is primarily eliminated by the kidneys (46).

SARS-CoV-2 infection promotes NT-proBNP expression, which is a marker of both cardiac injury and disease severity (47–49). Severe COVID-19 cases display a mean NT-proBNP level of 791 pg/mL, while milder cases have a mean of 160 pg/mL (49). Thus, NT-proBNP levels can be used as a biomarker of cardiac involvement and a prognosis indicator (50). The ventricular myocardium is the primary source of BNP. SARS-CoV-2 infection also causes increased hs-cTn and D-dimer levels, the degree of which is strongly correlated with poor prognosis in hospitalized patients (48, 51). The magnitude of hs-cTn and D-dimer elevation correlates with the final clinical outcome, ranging from hospital discharge to death.

During the SARS-CoV-2 pandemic, hospitalizations for myocarditis increased by 42.3%, compared with the prepandemic period. The risk for myocarditis was 0.146% among patients diagnosed with SARS-CoV-2 during an inpatient or hospital-based outpatient encounter and 0.009% among patients who did not have a confirmed case of SARS-CoV-2 infection. After adjusting for individual and local care factors, the adjusted risk of myocarditis among SARS-CoV-2 carriers was 15.7 (confidence interval, 14.1–17.2) times higher than that of SARS-CoV-2 negative carriers (52).

Endomyocardial biopsy is the gold standard for diagnosing acute and chronic inflammatory cardiomyopathies. Myocardial biopsies are accepted by the European Society of Cardiology (53) as gold standard investigative procedures for patients with myocarditis, using histochemical and viral genome analysis. A major drawback of the SARS-CoV-2 pandemic pertained to the technical difficulties imposed by performing these types of procedures in such conditions. However, magnetic resonance imaging can be a useful diagnostic resource for the identification of patients with cardiac involvement due to viral infection. Magnetic resonance imaging allows the detection of myocardial edema, hyperemia, necrosis, and/or fibrosis (Lake Louise criteria) (54, 55) with perfect correlation with the histological evidence of inflammation observed with endomyocardial biopsy (56–59).

Cardiovascular magnetic resonance imaging (CMR) is useful for the characterization of the myocardial tissue *in vivo*, providing insights into the pattern and degree of cardiac injury. In patients with SARS-CoV-2, the prevalence of myocardial involvement identified using CMR ranges from 26% to 60%; this variability is attributed to differences between populations, severity of illness, and interval between acute infection and CMR evaluation. The European Society for Cardiovascular Magnetic Resonance Imaging recommends CMR and provides recommendations for its use and reporting metrics, toward improved standardization, uniform data acquisition, and analytical approaches in patients with SARS-CoV-2 infection (56).

### 3.4. Heart failure and cardiogenic shock in SARS-CoV-2 infection

Heart failure can occur at different stages of SARS-CoV-2 infection, making it particularly challenging to diagnose and manage. Patients with heart failure alone have a higher chance of contracting SARS-CoV-2 infection because of their weakened immune systems, overall fragility, and reduced hemodynamic tolerance to dangerous infectious processes. Inflammatory cytokine generation, macrophage recruitment, and granulocyte release result in a severe inflammatory storm and increased metabolic demand, leading to acute or chronic decompensation and exacerbation of latent clinical illness. Other contributing factors are as follows: the occurrence of coagulation issues and thrombotic events, which are also associated with renal involvement in 15%–25% of SARS-CoV-2 infections and exacerbate cardiac and renal dysfunction (60); and increased sympathetic activity, which creates an imbalance between energy supply and consumption.

The most prevalent cardiovascular phenotype in hospitalized patients with SARS-CoV-2 infections is acute decompensated heart failure, which is characterized by severe congestion, drastically altered hemodynamic state, and increased biomarkers of myocardial injury (61). Up to 25% of hospitalized patients with COVID-19 develop new cases of heart failure. This complication is presumed to be a direct consequence of the virus or due to systemic inflammation. This causes acute myocarditis and, in some cases, results in cardiogenic shock, dysfunction of multiple organs, and death (62).

Different mechanisms can lead to cardiogenic shock, including fulminant myocarditis, which causes sudden hemodynamic impairment, global hypokinesia, biventricular dysfunction, hypotension, and multiple organ dysfunction syndrome (63). Cardiogenic shock can also be associated with type 1 infarction, in cases of patients with large infarction extension that progresses to the most severe classification on the Killip scale and with mechanical complications.

### 3.5. Isolated right ventricular failure

Right ventricular failure results from the advancement of pulmonary illness, cytokine production, and inflammatory interstitial pneumonia, leading to severe pulmonary embolism or microembolization. Patients may develop secondary right ventricular failure caused by mechanical ventilation–induced pulmonary injury and right ventricular systolic dysfunction (due to precapillary pulmonary hypertension resulting from pulmonary hypoxemia vasoconstriction). In patients with SARS-CoV-2 infection, acute myocarditis or a hypertensive emergency may contribute to the occurrence of right ventricular failure. Sudden changes that can contribute to this complication include the infectious process and hydrostatic modifications with increased capillary permeability and accumulation of fluid in the extravascular space, leading to alveolar edema (64).

### 3.6. Heart failure with reduced ejection fraction

Patients with SARS-CoV-2 may develop heart failure with reduced ejection fraction; however, its prevalence remains unclear. Further studies are needed, specifically those including outpatient follow-up, to help uncover the clinical cause of the infection and lingering cardiac involvement (61).

The primary causes of respiratory failure can be distinguished using biomarkers of cardiac injury, such as natriuretic peptides, in diagnostic imaging, which can also help determine the appropriate therapeutic approach.

## 4. Diagnostic imaging

To diagnose primary, secondary, or exacerbated cardiovascular problems linked to SARS-CoV-2 infection, conventional transthoracic echocardiography can be used (65, 66). In a recent report, although ventricular abnormalities were observed in 39% of patients with SARS-CoV-2 infections, examinations were normal in 45% of those patients (67). Of these, 3% had acute myocardial infarction, 3% had myocarditis, and 2% had Takotsubo disease. Left ventricular function deficits were classified as discrete, moderate, or severe in 17%, 12%, and 9% of patients, respectively. Similarly, 33% of the patients examined had functional alterations in the right ventricle. Discrete or moderate impairment was reported in 19% of patients, severe impairment in 6%, right ventricular dilation in 15%, and pulmonary hypertension in 8%. Both tamponade and endocarditis were detected only in 1% of patients. Furthermore, echocardiographic wall abnormalities were associated with well-defined clinical manifestations, such as chest pain with ST-segment elevation in 71% of the patients, elevated troponin and natriuretic peptides in 69%, suspected left or right ventricular failure in 60% each, and other alterations in 72% of the patients examined (68).

Nuclear magnetic resonance (NMR) is the gold standard imaging modality for the assessment of myocardial structure and function and simultaneous composition of myocardial tissue (56). NMR examination detects acute ischemic involvement (myocardial infarction type 1), non-ischemic myocardial injury (myocarditis), stress cardiomyopathy, acute heart failure, and secondary myocardial injury caused by sepsis or critical illness (69). The most frequent NMR parameters are T1-weighted images for representing myocardial anatomy with postadministration of gadolinium, demonstrating the distribution of contrast in the tissue, which is evidence of the existence of chronic lesions, and differentiating myocardial scar fibrosis in patients with SARS-CoV-2 infection. Native T1 mapping without gadolinium allows the detection of increased interstitial space (e.g., collagen accumulation or amyloid deposits) or increased intracellular and/or extracellular space (tissue water, i.e., myocardial edema) (54).

Necrosis/non-ischemic scarring involving the middle myocardium or epicardium can be detected using late-enhancement images, 10–15 min after gadolinium injection. These images show typical subendocardial infarct or scar involvement in the region of an obstructed coronary artery.

T2-weighted images with intense signal elevation are characteristic of tissue edema and associated with local inflammation; T2 mapping with increased time allows the detection of myocardial edema (70). A non-ischemic pattern during late gadolinium enhancement is usually linked to an abnormal T1 appearance and native T1, indicative of pericarditis or myopericarditis in patients with SARS-CoV-2. In patients with a high pretest probability of acute myocardial lesion–type myocarditis, magnetic resonance imaging can increase diagnosis sensitivity, facilitate treatment, and allow safe follow-up (69).

### 4.1. Electrocardiographic alterations in SARS-CoV-2

In this regard, ECG is an excellent research tool for detecting myocardial ischemia because it is a straightforward, accessible, affordable, and low-risk procedure (71).

Plaque rupture, coronary spasm, microthrombosis, endothelial dysfunction, hypoxia, electrolyte changes, and cytokine storms contribute to ECG changes during SARS-CoV-2 infection (72). These ECG alterations occur in 93% of patients hospitalized in the intensive care unit (ICU) with SARS-CoV-2 infection, which highlights the frequent comorbidity between COVID-19 and various arrhythmias and ECG alterations, as will be discussed in the following sections.

The main ECG alterations (73–78), molecular mechanisms of cardiac arrhythmias, and echocardiographic findings observed in patients with SARS-CoV-2 infection are presented in Supplementary Material Figure S2.

## 5. Arrhythmias

### 5.1. Supraventricular tachycardia

The most prevalent supraventricular arrhythmia in patients with SARS-CoV-2 infections is sinus tachycardia, which likely results from hypovolemia, hypoperfusion, hypoxia, and high body temperature. Conversely, AF is the most prevalent type of arrhythmia in patients with SARS-CoV-2-induced inflammatory cardiomyopathy. AF has variable presentations, such as sudden onset, recurrent pre-existing arrhythmia, and persistent or permanent AF with a rapid ventricular response, all of which are predictors of poor disease prognosis (79). Other arrhythmias that have been observed include atrial flutter, atrioventricular nodal reentrant tachycardia, and atrioventricular reentrant tachycardia, which are more common in younger individuals.

### 5.2. Malignant ventricular arrhythmias

Viral cardiomyopathy usually manifests as malignant ventricular arrhythmias, such as ventricular tachycardia and ventricular fibrillation in patients with SARS-CoV-2 infection. These arrhythmias can be caused by metabolic disorders or administration of drugs that prolong the rate-corrected QT (QTc) interval in the ECG. Monomorphic ventricular tachycardia has been observed in patients with structural myocardial diseases, such as acute coronary syndrome, ST-segment elevation myocardial infarction, and myocarditis. Polymorphic tachycardia, including torsade de pointes, results from functional heart diseases, such as drug toxicity, long QT, and Brugada syndrome (80).

### 5.3. Bradyarrhythmia and atrioventricular blocks in SARS-CoV-2

Ventricular atrial blocks occur less frequently than tachyarrhythmias. When patients with SARS-CoV-2 infection develop this type of block, an artificial cardiac pacemaker may be required. Cardiac arrest in these patients may be preceded by sinus bradycardia, nodal rhythm, or ventricular tachycardia; thus, bradycardia may be an indicator of cardiovascular collapse risk in patients with SARS-CoV-2 (80).

### 5.4. QT interval and other alterations in SARS-CoV-2

Patients with SARS-CoV-2 infection frequently present prolonged QT intervals, which is a cause for concern, as these can lead to malignant ventricular arrhythmias and cardiovascular death. At the beginning of the pandemic, a marked prolongation of the QT interval was observed in critically ill patients in ICUs receiving adjuvant therapies, including hydroxychloroquine, with or without concomitant azithromycin (81). This change in the ECG is associated with increased disease severity, serious cardiac injuries, and high mortality rates (82–84). However, a variety of factors can contribute to cardiac repolarization changes and impact the QT interval (85, 86). These include hereditary and acquired factors such as inflammatory processes, medications, treatments, and electrolyte imbalance.

ECG is a readily accessible tool that identifies cardiac involvement and can be used to predict the underlying cause of a disease (87). QRS and QTc intervals are early markers of SARS-CoV-2 disease progression and mortality (88). The exact mechanism by which SARS-CoV-2 infection may induce cardiac conduction abnormalities remains unknown. QT alterations may represent a simple marker reflecting the inflammatory state at the myocardial cellular level of the myocardium (83). The prolonged QTc intervals in ECG may result from the immune-mediated phenomena elicited by the virus infection, involving a cytokine storm with an elevation of IL-6 (89, 90), which blocks the potassium-related ether-a-go-go channel, contributing to increased circulating levels of IL-6.

QTc prolongation is likely more than just a drug-related side effect because the administration of drugs that extend the QTc interval does not impact the hospital mortality in patients with COVID-19 (83, 91). Jiménez-Jáimez et al. (92) analyzed 219 patients with ECG on admission. They reported that outpatients not critically ill with SARS-CoV-2 treated with hydroxychloroquine, azithromycin, and antiretrovirals developed a non-relevant prolongation of the QT interval (92). Significant waves in V1 and V2, ST-segment depression, T-wave inversion in leads II, III, arteriovenous fistula, V1 to V4, right and left branch blocks, and QRS axis deviations were indicative of right ventricular overload (93). Therefore, SARS-CoV-2 can have a deleterious effect on the cardiac conduction system, leading to significant ECG changes (79, 94).

### 5.5. Molecular mechanisms of cardiac arrhythmias in SARS-CoV-2

In SARS-CoV-2 infection, there are numerous possible mechanisms that increase the risk of cardiac arrhythmias. These include various forms of myocardial damage and extracardiac processes that may exacerbate arrhythmias in patients with a pre-existing propensity.

Myocarditis can cause arrhythmia in the acute phase as a direct cytopathic effect, resulting in electrical imbalance, ischemia (due to microvascular dysfunction), and junction dysfunction resulting from an impaired myocardial expression of connexins or ion channels. This is particularly common in patients with channelopathies with superimposed inflammation. Viral infections and host-related factors can alter the structural and electrophysiological properties of the myocardium in viral myocarditis, resulting in abnormal calcium movement and a negative regulation of potassium channels, leading to prolonged repolarization and abnormal conduction. Prolonged repolarization can induce deflagrated electrical activity in association with abnormal conduction (reduced conduction velocity and decreased refractoriness). Arrhythmias can also occur in the postinflammatory phase, in which variable degrees of myocardial scarring may exist, thereby promoting reentrant arrhythmias (95).

The systemic inflammatory response syndrome causes indirect myocardial damage. The intensive release of cytokines and chemokines, especially IL-1, IL-6, and TNF-α, is caused by a combination of micro- and macrovascular dysfunction, enhanced thrombogenicity, acidosis, hypoxia, and an imbalance in T-helper 1 and 2 responses. This process is amplified by enhanced catecholaminergic reactions; hyperinflammation due to high IL-6 levels results in a blockade of the hERG potassium channel and lengthening of the QT interval, facilitating the formation of unstable arrhythmias (96).

Inflammatory cytokines are well-studied triggers of arrhythmia, particularly in patients with a long QT syndrome, in which the cardiac sympathetic nervous system is overstimulated by the hypothalamus-mediated inflammatory reflex and peripherally mediated activation of the stellate ganglion pathway (96). Furthermore, IL-6 inhibits cytochrome P450, which increases the bioavailability of drugs that prolong the QT interval (97).

Hypoxia arising from lung injury or myocardial ischemia can activate anaerobic glycolysis, reducing intracellular pH, and thus increasing cytosolic calcium levels. This, in turn, can facilitate early and late depolarization and cause temporal changes in action potential duration. Hypoxia also increases extracellular potassium levels, which decrease the depolarization threshold and accelerate electrical conduction. In addition, hypoxemia can cause reduced electrical coupling and tissue anisotropy owing to the dephosphorylation of connexin 43 at communicating junctions (96).

In a previously published case series, the effects of electrolyte abnormalities on both preexisting and new arrhythmias were studied (3). These findings have been attributed to diarrhea associated with SARS-CoV-2 infection or renal injury (98), and severe electrolyte disorders, such as hypokalemia, hypomagnesemia, and hypophosphatemia, are also linked to atrial arrhythmias (99).

Traditional cardiovascular risk factors such as type II diabetes mellitus, hypertension, and hypercholesterolemia, as well as comorbidities such as ischemic heart disease and chronic renal failure, also contribute to the development of arrhythmia by altering the cardiac structure. Another potential contributor to the development of SARS-CoV-2 infection is the p.Ser1103Tyr variant of the common *SCN5A*-encoded Nav1.5 sodium channel that results in a lack of “repolarization reserve” (91).

## 6. Epidemiological changes in SARS-CoV-2 infection variants

Three epidemic waves have occurred since the first SARS-CoV-2 wave in March 2020, with the second and third waves dominated by the beta (B.1.351) and delta (B.1.617.2) variants, respectively. The fourth pandemic wave was caused by variation B.1.1.529, which the Network for Genomic Surveillance in South Africa identified as Omicron on 24 November 2021, the fifth variant of concern. This variant demonstrated a 70% decreased propensity to cause severe disease and thus lower hospitalization rates than the delta variant (100). In contrast to earlier waves, the early phase of the fourth wave in South Africa showed a different pattern of disease features and outcomes, with younger patients exhibiting fewer comorbidities, hospitalizations, and respiratory diagnoses, as well as a decline in severity and mortality. Despite this reduction in the pathogenicity of the Omicron variant, further research is required to determine the roles of acquired (vaccination) or natural immunity in the pandemic waves (101).

Another aspect that should be considered is the efficacy of SARS-CoV-2 vaccines against infection. Efficacy decreased considerably 5–8 months after primary vaccination, although it remained high, particularly among those under 55 years of age. Nevertheless, vaccine boosters were effective in restoring protection against infection and had a good safety profile in the community, which contributed to the reduction in the severe consequences of SARS-CoV-2 infection (102).

### 6.1. Long-term cardiovascular sequelae and cardiovascular risk modification after SARS-CoV-2 infection

Most research on cardiovascular outcomes during the acute stage of hospitalization, which represents a minority of patients infected with SARS-CoV-2, does not adequately address the long-term cardiovascular sequelae of the infection. However, a database analysis (103) of cardiovascular outcomes after a 12-month follow-up revealed that hospital readmission is associated with a high mortality rate, with multiple organ dysfunction being the primary cause (104). Nevertheless, a persistent elevation of myocardial injury biomarkers, such as hs-cTn, NT-proBNP, and D-dimer, indicates injury or underlying heart disease, and an increased risk of myocarditis, pericarditis, coronary artery disease manifested by acute coronary syndrome, myocardial infarction, angina, and ischemic cardiomyopathy. Other symptoms include heart failure, non-ischemic cardiomyopathy, left ventricular systolic and diastolic dysfunction, deep vein thrombosis, and pulmonary thromboembolism (105).

Also, the post-SARS-CoV-2 group also displays an increased incidence of AF, sinus tachycardia and/or bradycardia, ventricular arrhythmias, and atrial flutter.

In patients with SARS-CoV-2 infection, 12 months after hospital release, there is an increased risk of developing diabetes mellitus, and some prediabetic individuals progress to diabetes after infection (106). One hypothesis is the expression of ACE2 in pancreatic islet cells, where it could promote direct viral infiltration, resulting in inflammation and loss of pancreatic beta cells, and contributing to the development of diabetes mellitus (107).

There is also evidence suggesting a higher risk of developing hypertension in individuals post-SARS-CoV-2 infection, as well as poorer blood pressure control. Although the mechanisms are not fully understood, ACE2 may be involved in reducing the renin–angiotensin–aldosterone system by converting angiotensin 1 and 2 into angiotensin 1–9 and 1–7, respectively. This is accompanied by an increased bioavailability of angiotensin 2 and a subsequent increase in blood pressure (108). Inadequate control of blood pressure in the postinfection stage is suggestive of lifestyle changes; a decrease in physical activity, unhealthy diets, and increased psychosocial stress have been observed as a result of the pandemic (109).

Finally, a prolonged clinical picture of symptoms ranging from weeks to months post-SARS-CoV-2 infection called “long SARS-CoV-2” has been observed, which includes a broad spectrum of symptoms such as fatigue, exertional dyspnea, chest pain, palpitations, headache, nausea, vomiting, skin rashes, joint pain, anxiety, and depression. Although there is no standard universal criterion to characterize this condition, the World Health Organization has proposed that long SARS-CoV-2 should be defined as clinical manifestations lasting more than 3 months and symptoms lasting ≥ 2 months not explained by another disease (110, 111).

The goal of this mini review was to provide an overview of the literature on the relationship between SARS-CoV-2 infection and the clinical manifestations of cardiovascular system involvement in children and adults, including important disorders affecting cardiac rhythm. We summarized the potential mechanisms that could be involved in the expression of the different clinical manifestations and the mechanisms underlying the complications of arrhythmias associated with SARS-CoV-2 infection and discussed the main imaging methods that allow appropriate diagnoses to be made.

## 7. Conclusions

Since the beginning of the pandemic, the medical and scientific communities have made enormous efforts to detect the early clinical cardiovascular manifestations caused by SARS-CoV-2 infection.

Children with cardiovascular system involvement display a clinical profile similar to that of patients with Kawasaki disease, including heart failure and coronary artery involvement. In adults, its main clinical manifestations are coronary artery disease, stress-induced cardiomyopathy, myocarditis, heart failure, and arrhythmias, some of which are benign, such as transient sinus bradycardia, or potentially fatal, such as ventricular tachycardias and torsade de pointes, leading to sudden death. AF is the most prevalent arrhythmia in critically ill patients with SARS-CoV-2 infection with fibrillation. Owing to the severity of the infection and the concurrent use of proarrhythmogenic antimicrobials and anti-inflammatory medications, the management of these arrhythmias requires special consideration.

Imaging techniques, such as conventional transthoracic echocardiography and ECG, are used to assess the cardiological manifestations of SARS-CoV-2 infection and diagnose primary, secondary, or associated cardiovascular complications. NMR is the most accurate technique for assessing myocardial structure and function. Cardiac enzymes hs-cTn and NT-proBNP can be used to detect cardiac involvement and determine prognosis.

Comorbidities, such as hypertension, diabetes mellitus, and coronary heart disease, are risk factors for patients with COVID-19 that can aggravate the clinical course of the disease.
