# Supplementary figures and images for: Pathophysiological effects of SARS-CoV-2 infection on the cardiovascular system and its clinical manifestations—a mini review
Source: Front Cardiovasc Med. 2023 May 16;10:1162837. doi: 10.3389/fcvm.2023.1162837 (PMC10229057; doi:10.3389/fcvm.2023.1162837)

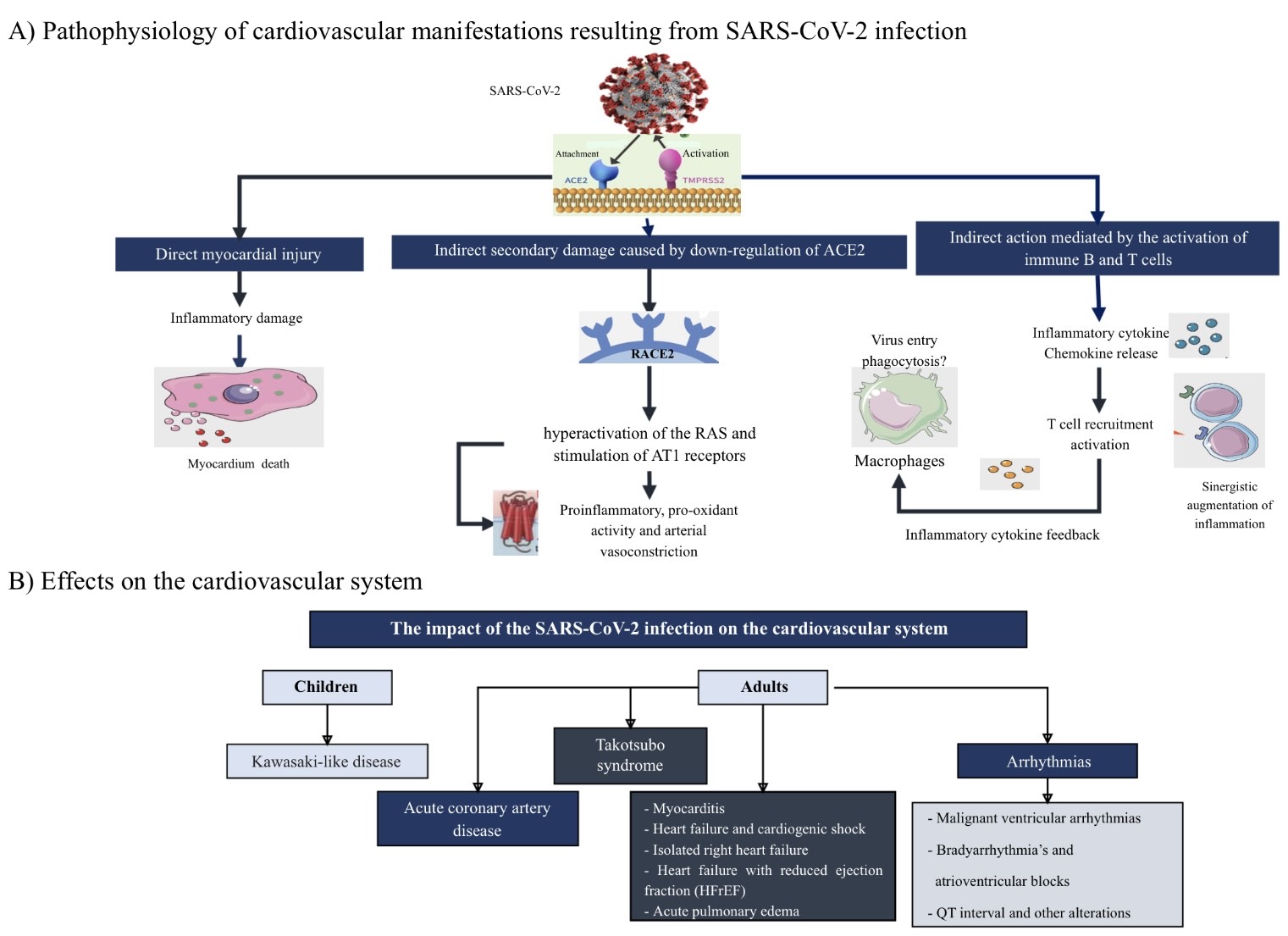

Supplement: Supplementary file 1 [file Image1.jpg]

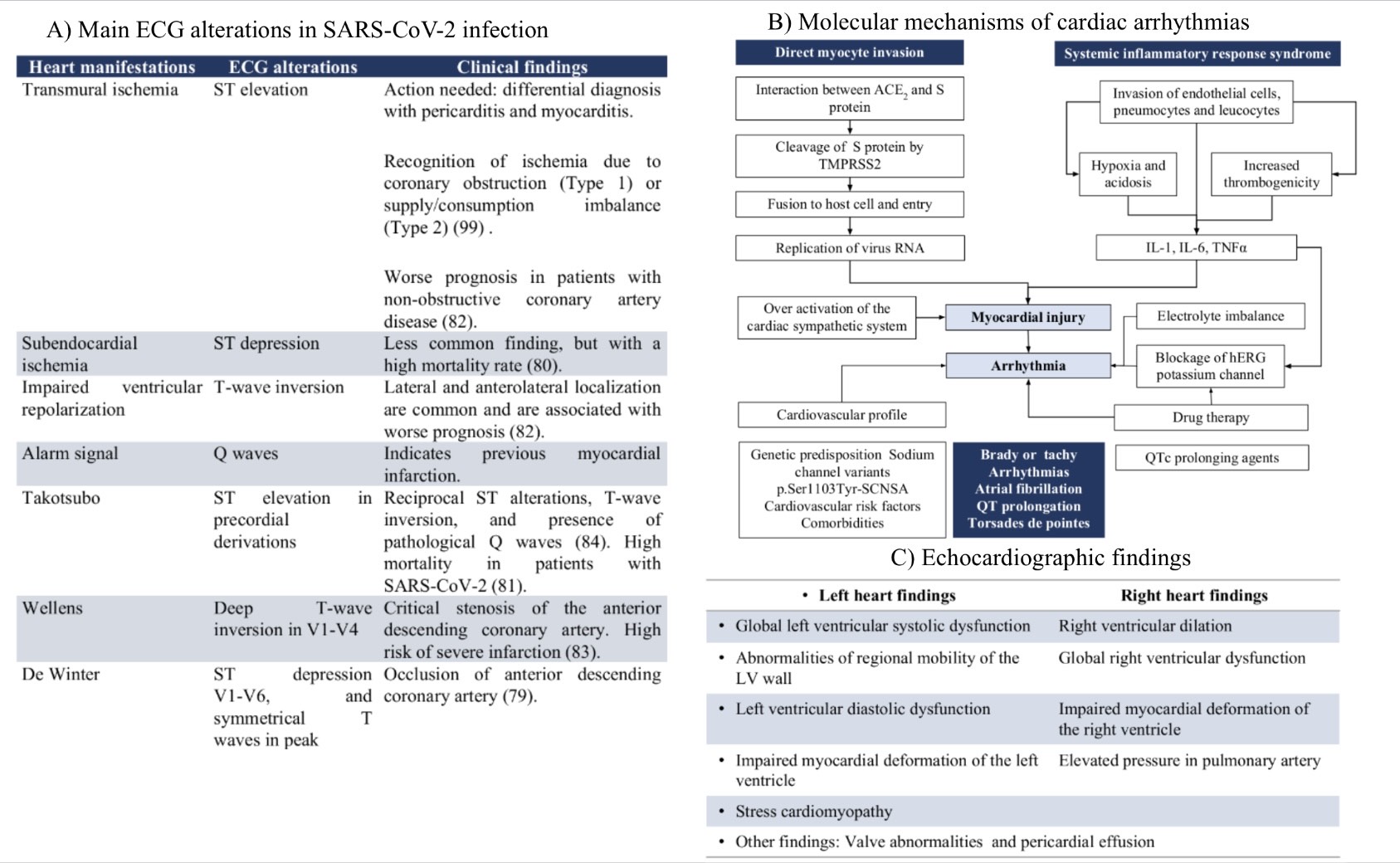

Supplement: Supplementary file 2 [file Image2.jpg]
